# Comparison of the Behavior of 3D-Printed Endothelial Cells in Different Bioinks

**DOI:** 10.3390/bioengineering10070751

**Published:** 2023-06-23

**Authors:** Jana Schulik, Sahar Salehi, Aldo R. Boccaccini, Stefan Schrüfer, Dirk W. Schubert, Andreas Arkudas, Annika Kengelbach-Weigand, Raymund E. Horch, Rafael Schmid

**Affiliations:** 1 Laboratory for Tissue-Engineering and Regenerative Medicine, Department of Plastic and Hand Surgery University Hospital of Erlangen, Krankenhausstraße 12, 91054 Erlangen, Germany; 2 Chair of Biomaterials, University of Bayreuth, Prof.-Rüdiger-Bormann-Str. 1, 95447 Bayreuth, Germany; 3 Institute of Biomaterials, Friedrich-Alexander University Erlangen-Nürnberg, Cauerstrasse 6, 91058 Erlangen, Germany; 4 Institute of Polymer Materials, Friedrich-Alexander University Erlangen-Nürnberg, Martensstraße 7, 91058 Erlangen, Germanydirk.schubert@fau.de (D.W.S.)

**Keywords:** endothelial cells, bioprinting, tissue engineering, vascularization, biofabrication, hydrogels

## Abstract

Biomaterials with characteristics similar to extracellular matrix and with suitable bioprinting properties are essential for vascular tissue engineering. In search for suitable biomaterials, this study investigated the three hydrogels alginate/hyaluronic acid/gelatin (Alg/HA/Gel), pre-crosslinked alginate di-aldehyde with gelatin (ADA-GEL), and gelatin methacryloyl (GelMA) with respect to their mechanical properties and to the survival, migration, and proliferation of human umbilical vein endothelial cells (HUVECs). In addition, the behavior of HUVECs was compared with their behavior in Matrigel. For this purpose, HUVECs were mixed with the inks both as single cells and as cell spheroids and printed using extrusion-based bioprinting. Good printability with shape fidelity was determined for all inks. The rheological measurements demonstrated the gelling consistency of the inks and shear-thinning behavior. Different Young’s moduli of the hydrogels were determined. However, all measured values where within the range defined in the literature, leading to migration and sprouting, as well as reconciling migration with adhesion. Cell survival and proliferation in ADA-GEL and GelMA hydrogels were demonstrated for 14 days. In the Alg/HA/Gel bioink, cell death occurred within 7 days for single cells. Sprouting and migration of the HUVEC spheroids were observed in ADA-GEL and GelMA. Similar behavior of the spheroids was seen in Matrigel. In contrast, the spheroids in the Alg/HA/Gel ink died over the time studied. It has been shown that Alg/HA/Gel does not provide a good environment for long-term survival of HUVECs. In conclusion, ADA-GEL and GelMA are promising inks for vascular tissue engineering.

## 1. Introduction

As cardio-vascular diseases remain one of the major causes of mortality and morbidity, especially in the context of an ever-aging population, the need for vascular replacement and, ideally, the advent of engineered blood vessels as well as vascular prostheses is increasing. Currently, the options for vascular grafts are still very limited. On the one hand, alloplastic substitutes suffer from a short patency rate, and allogeneic and xenogeneic vessel grafts are immunologically rejected and may hence lead to severe problems, including shrinkage with postoperative stenosis, so the demand for advanced engineered grafts is enormous [1]. However, even synthetic grafts still have shortcomings. Therefore, to overcome these limitations, researchers are constantly searching for new biomaterials and new compositions of materials [2]. The main goal for suitable biomaterials is to mimic the natural tissue to achieve functional and structural tissue formation [3]. Therefore, the ideal condition for cell growth and angiogenesis would be an extracellular matrix (ECM), as found in the human body [4]. To this end, sophisticated in vivo experiments are performed to analyze the angiogenesis of implanted constructs [5]. The ECM is composed of various types of proteins and glycans. It provides structural and biochemical support for cell growth and provides cells with biochemical and biomechanical stimulation so that tissue differentiation and the development of organelles are triggered [3,6]. Even though the building blocks of ECM all have very high biocompatibility, they cannot be manufactured as precisely as synthetic polymers [4]. In 3D bioprinting applications, hydrogel-based inks are used as a matrix to build scaffolds and can be originally composed of synthetic or natural polymers. Synthetic polymers are easy to handle and can tailor their properties to specific applications. However, they are often poorly biocompatible; therefore, natural-based hydrogels are favored. Hence, the inks used for 3D bioprinting applications must be printable and biocompatible and should also possess suitable mechanical and structural properties [1]. Hydrogels are hydrophilic, polymeric networks that can absorb large amounts of water and, due to their soft, rubbery consistency, provide a tissue-like environment for the encapsulated cells [7]. Hydrogels based on alginate dialdehyde (ADA) are ideally suited as materials for tissue engineering due to their biocompatibility, biodegradability, and rapid degradation rates [8]. Since alginate is biocompatible and rapidly ionically gelled, it is suitable for the encapsulation of cells and for biofabrication. The use of alginate is limited by insufficient material-cell interaction and inefficient cell adhesion. However, the incorporation of gelatin by covalent crosslinking with alginate dialdehyde (ADA) can overcome these limitations. Gelatin, which is present in ADA-GEL, is a biodegradable protein produced by acidic or basic hydrolysis of collagen [9]. Gelatin is particularly well suited for vascular tissue engineering because it possesses certain properties that facilitate angiogenesis [10]. For example, gelatin contains integrin-binding motif (RGD) sequences that enable endothelial cells to degrade the hydrogel, migrate, spread, and provide adequate cell attachment [10,11]. As gelatin is produced from collagen, it performs similar cell functions and is also important for cell proliferation and differentiation [12,13]. The potential of ADA-GEL for tissue engineering and biofabrication has already been demonstrated [14,15].

In 2021, Schmid et al. (2021) [7] reported another alginate-gelatin-based ink, which consists of the three components alginate, hyaluronic acid (HA), and gelatin (Alg/HA/Gel). While alginate is a polysaccharide derived from brown algae, HA is a natural glycosaminoglycan found in almost all connective tissues. Since HA is a natural extracellular matrix material that is naturally biocompatible and has water-binding properties, it is suitable as a biopolymer for tissue engineering. HA plays an important role in many cell activities and tissue functions in the body, such as cell migration, proliferation, differentiation, and angiogenesis [16]. The newly developed Alg/HA/Gel ink has been demonstrated to have good printability, high shape fidelity, and high tumor cell survival [7].

In addition, there are also gelatin-based inks that have been modified. For example, the biodegradation of gelatin can be adapted by functionalization with methyl acrylate (MA), resulting in gelatin methacryloyl (GelMA). Since crude gelatin forms a hydrogel, which has low mechanical strength and is liquid in cell culture, a crosslinking chemical is used to improve stiffness [17,18]. The formation of covalently crosslinked hydrogels is achieved by a photoinitiator system under mild conditions, which triggers the formation of free radicals that polymerize the various methacrylamide and methacrylate groups inside the gelatin. Light exposure of the ink in the presence of a photoinitiator increases the stability of the hydrogel by the formation of irreversible chemical crosslinks between the protein chains and results in the encapsulation of the cells, which allows them to be highly viable [10,11,19]. The encapsulation of HUVECs in GelMA as well as the use of GelMA for soft tissue engineering applications have already been successfully demonstrated in the literature [10,11]. In contrast, the encapsulation of HUVECs in the form of single cells and spheroids in the hydrogels Alg/HA/Gel and ADA-GEL for bioprinting has been described little, if at all, in the literature [20]. Therefore, in the following study, small scaffolds from the three inks described were printed incorporating HUVECs with an extrusion-based bioprinter. Extrusion-based bioprinting offers the advantage over conventional hydrogel models that hydrogels and cells can be combined at different concentrations to create macroporous structures and complex 3D architectures [7,21] and can be used for vascular models [22]. Furthermore, extrusion-based bioprinting is characterized by its simplicity, affordability, accuracy, and reproducibility [23].

Here, endothelial cells were printed both as single cells and as cell spheroids in the different hydrogels to create a 3D microenvironment. The mechanical properties, such as the stiffness and degradation behavior of the inks, were analyzed. With respect to a successful printing process, the rheological properties and the printability of the inks were further investigated. To study the behavior of endothelial cells in different inks, single cells were observed for 14 days for their metabolic activity, cell survival, and alignment. The spheroids were studied for 7 days in terms of sprouting, migration, survival, and alignment. To optimally compare the behavior of the cells in the printed constructs, both the single cells and the spheroids were additionally printed in Matrigel.

## 2. Materials and Methods

### 2.1. Cell Culture

HUVEC hTERT2 (Evercyte GmbH, Vienna, Austria) cell line was cultured in 500 mL Endothelial Cell Growth Medium-2 (EGM-2) from Lonza Clonetics (Basel, Switzerland) after addition of various supplements. A total of 50 mL FCS superior (standardized fetal bovine serum, Biochrom GmbH, Berlin, Germany), 2.0 mL recombinant human fibroblast growth factor-B (rhFGF-B), 0.5 mL recombinant insulin-like growth factor (R^3^-IGF-1), 0.5 mL recombinant human epidermal growth factor (rhEGF), 0.5 mL vascular endothelial growth factor (VEGF), 0.5 mL heparin, 0.5 mL ascorbic acid, 0.5 mL geneticin (final concentration 20 µg/mL, Thermo Fisher Scientific, Waltham, MA, USA), and 0.2 mL hydrocortisone were added. The incubator settings were 5% CO_2_ and 37 °C. The medium was changed three times per week for constant nutrient supply.

### 2.2. Production of the Bioinks

#### 2.2.1. ADA-GEL

The ADA-GEL was prepared following the protocol of Hazur et al. (2020) [24]. In the first step, 2 mL of a 6.25 wt.% stock solution of ADA (13% degree of oxidation) (prepared from Alginate VIVAPHARM^®^ PH 163 S2 JRS PHARMA GmbH & Co. KG, Rosenberg, Germany) was prepared. For this, 0.125 g of ADA and 2 mL of Dulbecco’s Phosphate-buffered saline (PBS, Sigma-Aldrich, St. Louis, MO, USA) were homogeneously dissolved in a 50 mL beaker with constant stirring. Meanwhile, a second solution consisting of 6.25 wt.% gelatin (Sigma-Aldrich, St. Louis, MO, USA) in PBS containing 250 mmol/L CaCO_3_ (Calcium carbonate precipitated for analysis, EMSURE^®^, Merck KGaA, Darmstadt, Germany) was prepared. For this, 0.188 g gelatin was dissolved in 3 mL PBS on a hot plate with constant stirring. Once the gelatin dissolved completely, 75 mg of CaCO_3_ was added, and the solution was homogenized for an additional 10 min. Once the stock solution containing the ADA-GEL dissolved completely, 2 mL of the second prepared gelatin/CaCO_3_ solution was added using a micropipette and stirred for another 20–30 min at 37 °C. Meanwhile, the third and final solution was prepared. To prepare 2 mL of a 250 mmol/L D-Glucono-δ-lactone (GDL, Sigma-Aldrich, St. Louis, MO, USA) solution, 2 mL of ultrapure water containing 89 mg GDL was stirred for 1 min. The solution should be prepared just before the mixing step. An amount of 1 mL of the GDL solution was added dropwise to the ADA-GEL/CaCO_3_ mixture, and the entire mixture was stirred for 3 h at 37 °C. After the ink has been stirred, it can be carefully transferred with the cells into a cartridge for 3D printing.

#### 2.2.2. Alg/HA/Gel

The ink was prepared according to the protocol of Schmid et al. (2021) [7]. The ink consists of 3 wt.% gelatin (Sigma-Aldrich, St. Louis, MO, USA), 0.5 wt.% alginate (Alginate PH 176, JRS Pharma GmbH &Co. KG), and 0.1 wt.% HA (high molecular weight hyaluronic acid 1–2 MDA, CarboSynth Ltd., Compton, UK), all dissolved in PBS. 0.3 g of gelatin, 0.05 g of alginate, and 0.01 g of HA were weighed and dissolved in a beaker containing 10 mL of PBS at 37 °C on a magnetic stirrer for 1.5 h. The beaker was covered with a sealing film (PARAFILM^®^M, 100 mm, 75 m, Roth, Karlsruhe, Germany).

#### 2.2.3. GelMA

The GelMA used for the ink was synthesized according to the protocol described in Loessner et al. (2016) [19]. For this purpose, 6 g of gelatin A (porcine, Sigma-Aldrich, St. Louis, MO, USA) was dissolved in 50 mL PBS and mixed with 12 mL of methacrylic anhydride (MA, Sigma-Aldrich, St. Louis, MO, USA) with stirring at 50 °C for 1 h. The methacrylation process was terminated by adding warm PBS. The solution was then dialyzed at 37 °C for one week and lyophilized for another week. The obtained GelMA has an estimated degree of functionalization of about 80%. It was stored at −20 °C. To prepare the ink, 4 wt.% of the produced GelMA was dissolved in PBS. For this, 80 mg of GelMA was weighed and homogenized in a suitable beaker in 1.8 mL of PBS at 37 °C with constant stirring for 30 min. Under the exclusion of light, 10 mg of lithium phenyl-2,4,6-trimethylbenzoyl phosphinate (LAP, Sigma-Aldrich, St. Louis, MO, USA) was weighed, dissolved in PBS, and added to the homogenized GelMA solution to give a concentration of 0.1 wt.% LAP in the solution. To obtain complete homogenization of the solution with the added LAP, it was stirred for an additional 10–15 min under protection from light.

### 2.3. Preparation of Cells and Spheroids for the Bioprinting Process

#### 2.3.1. Single Cells

Single cells used for printing were detached from the T-75 cell culture flasks (TC-flask T75, Sarstedt AG & Co. KG, Nümbrecht, Germany) by first removing the medium and then washing the cells with PBS. The detachment process was initiated by adding accutase (Merck KGaA, St. Louis, MO, USA), which was incubated for 4 min. After incubation, the accutase cell suspension was diluted with PBS, transferred to a Falcon tube, and centrifuged. After centrifugation, the supernatant was aspirated, leaving the cells bundled as a pellet at the bottom of the Falcon tube (Falcon Conical Centrifuge Tubes, Corning, New York, NY, USA). A total of 5 × 10^6^ HUVEC cells were used per 1 mL ink.

#### 2.3.2. Spheroids

Spheroids were generated using the hanging drop method. For this, methylcellulose medium was first generated by autoclaving 6 g of methylcellulose (Sigma-Aldrich, St. Louis, MO, USA) in 500 mL bottles. Then, 250 mL of the basal medium was heated to 60 °C and added to the methylcellulose. The solution was stirred for 20 min at room temperature on a magnetic plate. After 20 min, another 250 mL of medium was added to the solution, and the entire solution was stirred at 4 °C overnight. The solution with a volume of 500 mL was transferred to ten 50-mL Falcons the next day and centrifuged at 3500× *g* at 4 °C for 300 min. The generated methylcellulose medium in the falcons was stored at 4 °C. To generate the hanging drops, the cells were detached as described in Section 2.3.1, and the cells in the Falcon were counted. 750 × 10^3^ cells were suspended in 20 mL of HUVEC cell culture medium and 5 mL of methylcellulose medium to obtain a total of 1000 spheroids with 750 cells each. Droplets with a volume of 25 μL were generated on rectangular Petri dishes using a multipipette. The Petri dishes were inverted and stored overnight at 37 °C in the incubator with 5% CO_2_. The next day, the spheroids could be harvested. For this, the spheroids were washed down from the Petri dishes with PBS and transferred to a Falcon. The Falcon was then centrifuged for 4 min at 300× *g* and 22 °C. The deceleration was set to zero. The supernatant was again aspirated.

### 2.4. Bioprinting Process

**Figure 1:**
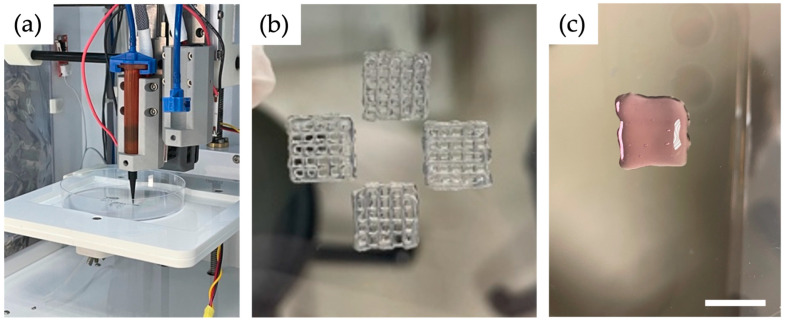
(**a**) Cartridge during the printing process with a black needle blocking the UV light. (**b**) Four scaffolds in a Petri dish after a successful printing process with ADA-GEL. Crosslinking took place afterwards. (**c**) One specimen printed with Matrigel. The insufficient shape fidelity is clearly visible. Since it is known that Matrigel does not have sufficient mechanical properties, these were also not analyzed further. Scale bars = 1 cm.

The Cellink Inkredible+ (Cellink, Boston, MA, USA) printer was used for printing. Using a displacement pipette, both cells (10 × 10^6^) and spheroids (1000 à 750 cells) were carefully resuspended with 2 mL of ink each and then transferred to a 3 mL cartridge for printing (Figure 1a). Subsequently, the gel-filled cartridge (GelMA and Alg/HA/Gel; ADA-GEL requires an intermediate step) was cooled in a water bath at 15 °C for 6 min to obtain adequate viscosity with a true-to-form print result. Before the cooling process, the cartridge was filled with ADA-GEL and cells and placed in the centrifuge. At 872× *g* for 7 min, the air bubbles were removed from the gel. Subsequently, the cartridge was also placed in the water bath at 15 °C for 6 min. A conical needle with an inner diameter of 0.41 mm and a length of 127 mm was used to print 1 cm^2^ scaffolds (grids with three layers). To print the GelMA ink a conical UV-restrictive needle of the same diameter, and a UV-restrictive cartridge was used. A pressure of 20–25 kPa was used for all inks. The pressure range results from the slightly fluctuating temperature within the printer. The ink in the needle was not cooled, unlike the ink in the cartridge. Therefore, after a print, the pressure had to be reduced slightly for a short time for the next printing process. Each scaffold consisted of three layers with six strands. Four scaffolds could be printed in a Petri dish in a row (Figure 1b). Printing the four scaffolds took about 15 min. The experiments could be performed in biological triplicates (n = 3).

The cells and the spheroids were also printed in the standard material Matrigel (Corning^®^ Matrigel^®^ basement membrane matrix, Corning Life Sciences, Tewksbury, MA, USA). For this purpose, the Matrigel was thawed at 4 °C overnight. The liquid Matrigel was carefully suspended with the cells and filled into the cartridge. To obtain an increased viscosity, the cartridge was left to gel at room temperature for about 10 min. After that, the Matrigel could be printed using a much lower pressure of 3–5 kPa (Figure 1c). To allow the Matrigel to gel completely, the scaffolds were subsequently placed in the incubator at 37 °C and 5% CO_2_ for 30 min before adding the medium.

### 2.5. Crosslinking

Alginate-based inks are usually crosslinked with divalent cations, such as Ca^2+^. A gel network is formed because one alginate chain dimerizes and forms a bond with many other alginate chains [25]. Since the pre-crosslinked ADA-Gel exhibits relatively rapid decomposition in the culture medium, it has been found useful to add microbial transglutaminase (mTG, Ajinomoto, Tokyo, Japan) to the CaCl_2_ solution for crosslinking. By adding mTG, a slower rate of degradation could be achieved [24,26]. To prepare the solution, 100 mM CaCl_2_ (Calcium chloride dihydrate, Merck KGaA, Darmstadt, Germany) was mixed with 5 wt.% mTG in a beaker and then added to the constructs for 10 min at room temperature. Alg/HA/Gel was crosslinked with 100 mM CaCl_2_ for 10 min after printing at room temperature. The GelMA bioink containing the photoinitiator LAP was crosslinked with a hand-held lamp (405 nm) for 30 s at a distance of 5–10 cm.

Construct Handling

All printed scaffolds (ADA-GEL, Alg/HA/Gel, and GelMA) were subsequently washed with medium and transferred to 6-well plates containing cell culture medium. The constructs containing single cells were incubated for 14 days and the spheroid-containing constructs for 7 days at 37 °C and 5% CO_2_. The medium was changed three times per week. To stimulate sprouting, phorbol 12-mristate 13-acetate (PMA, concentration 100 ng/mL, Abcam, Cambridge, UK) was also added to the medium for the spheroid-containing scaffolds. When PMA, which strongly stimulates the angiogenesis of HUVECs, is added at appropriate concentrations, it accelerates the migration and network formation of HUVECs [27].

### 2.6. Live/Dead Cell Assay

To perform the live/dead assay, staining was performed with calcein-acetoxymethyl (Sigma-Aldrich, St. Louis, MO, USA) and propidium iodide (Sigma-Aldrich, St. Louis, MO, USA). Staining of single cells was performed on days 1, 7, and 14, whereas spheroids were stained on days 0, 1, 2, 3, 4, and 7. For staining of three scaffolds, calcein-AM (2.01 µM) was put into the cell culture medium in a light-protected Falcon, and propidium iodide (1.5 µM) was homogenized with culture medium in another light-protected Falcon. The scaffolds were transferred to 24-well plates. An amount of 500 μL of the calcein-AM/medium mixture was added to each well containing one scaffold, and the plates were placed in the incubator for 30 min at 37 °C and 5% CO_2_. Then the calcein-AM solution was carefully aspirated, and 500 μL of the propidium iodide mixture was added to the scaffolds. The plates were incubated at room temperature in the dark for 5 min. Subsequently, the propidium iodide solution was aspirated, and 1 mL of Hank’s Balanced Salt Solution (HBSS, Sigma-Aldrich, St. Louis, MO, USA) was pipetted into the wells for washing for 15 min. After 15 min without exposure to light, the HBSS was removed and 500 μL of HBSS was added to the scaffolds. Then, the scaffolds were analyzed by fluorescent microscopy (Olympus IX-83, cellSens Software V1.16, Olympus Corporation, Tokyo, Japan). Three images per scaffold were taken according to a standardized procedure at the surface and 100 µm into the gel. Using the open-source software FIJI (Version: 2.9.0/1.53t), a distribution of ImageJ and the calcein-AM and PI fluorescence were merged for the assessment of the cells. Each staining was performed in technical triplicates. The spheroids were measured per biological replicate (n = 8) on days 1, 2, 3, 4, and 7. Staining of spheroids on day 0 was repeated two times, and two spheroids per hydrogel were recorded. Live and dead cells were counted manually. The length of the sprouts of spheroids was measured manually using the program FIJI, starting from the core of the cell. The three farthest migrated cells were identified, and the migratory distance was also measured using FIJI.

### 2.7. WST-8 Assay (Metabolic Activity Assay)

Cell proliferation is indicated by the reduction of the water-soluble tetrazolium salt WST-8 (2-(2-methoxy-4-nitrophenyl)-3-(4-nitrophenyl)-5-(2,4-disulfophenyl)-2H-tetrazolium, monosodium salt) of NADH to a yellow formazan dye that dissolves in tissue cultures. The enzyme NADH is active exclusively in living cells and is required for dehydrogenase activities in mitochondria [28,29]. The higher the cell number, the higher the activity of dehydrogenases, and the more dye can be produced [29]. The proliferation assay was performed on days 1, 7, and 14 of the single cells printed on scaffolds. For this, 500 μL of HUVEC cell culture medium and 50 μL of WST-8 (PromoCell, Heidelberg, Germany) were mixed in four wells of a 24-well plate, protected from light. Then, three scaffolds of a print were transferred into one well each, leaving one well as a blank reference. The 24-well plate was then placed in the incubator for 2 h. After 2 h, the absorbance of each sample was measured. For this, 100 μL was further taken from each of the four wells three times without exposure to light and transferred to one well of each 96-well plate. The 96-well plate was then placed in a photometer so that the amount of formazan could be quantified by measuring the absorbance at wavelengths of 450 nm and 600 nm as a background. Here, the level of absorbance provides information about the number of metabolically active living cells in the scaffold measured per biological replicate [29].

### 2.8. Printability Assay

**Figure 2:**
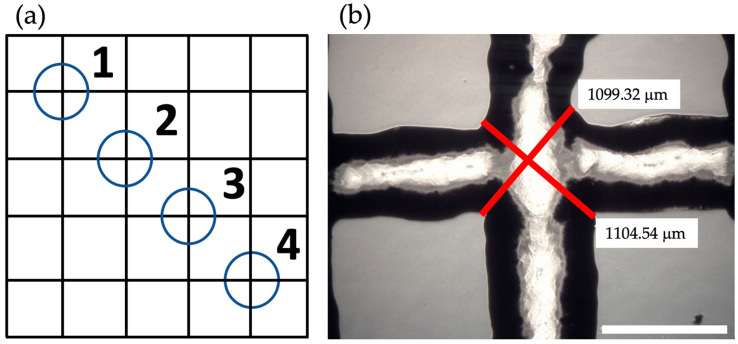
(**a**) Representation of a printed scaffold with four selected crossing points (adapted from Bednarzig et al. (2022) [30]). (**b**) Exemplary picture of a scaffold printed with ADA-GEL. Care was taken at the crossing point to ensure that the marked lines were always at right angles to each other. The shown lengths of the crossing lines were measured using the microscope. Scale bar = 1 mm.

Immediately after the printing process, the printability of the scaffolds was tested. The printed scaffolds were placed under a light microscope for optical magnification. Four different crossing points of the scaffold lines per scaffold were selected. The length of the lines at the crossing point was measured (see Figure 2a,b). The lines should always be perpendicular to each other, as would be the case in an ideal grid. The ideal length of the lines at the intersection of an ideal grid would be 579.828 μm due to the inner needle diameter of 410 µm. Using this ideal value, the diagonal crossing ratio (DCR) for the intersection points can be determined by dividing the ideal length of the line by the averaged real value of the lines [30]. The printability test was evaluated using 20 technical replicates.

### 2.9. Rheometer Measurements

A DHR-3 rheometer (TA Instruments, New Castle, DE, USA), equipped with a 20 mm plate-plate geometry, was used. A Peltier element (bottom plate) is used to ensure temperature control during all measurements. After the preparation, 200 μL of the uncured inks was added between the plate-plate geometry. Excessive material was removed after lowering the top plate to the desired measurement gap of 500 microns. The temperature of the rheometer was set at 37 °C. Then, the hydrogels were cooled down to 15 °C at 2 °C/min. The temperature was maintained for 6 min to ensure homogeneous temperature distribution in the inks. After that, the oscillation experiments were performed, in which the storage modulus G′ and the loss modulus G″ were measured. A temperature ramp and holding of the temperature for 6 min, two frequency sweeps at 10-min intervals with a constant oscillation displacement of 1% and an increasing frequency of 0.1–100 rad/s (0.016–16 Hz), and an amplitude sweep with a frequency of 10 rad/s and an amplitude between 0.035% and 110.5% were measured. From the measurement of the frequency sweeps 1 and 2, the values of complex viscosities 1 and 2 could also be determined. For better readability, the values of complex viscosity is referred to as complex viscosity in the following manuscript. Three technical replicates were composed from one batch of ink.

### 2.10. MicroTester LT Measurements

The Young’s modulus of the hydrogels was determined using the MicroTester LT from CellScale (Waterloo, ON, Canada). For the measurements, samples were cast from silicone molds with cylindrical recesses with a diameter of 5 mm. An amount of 40 μL of the ink was used per cavity. Afterwards, the inks were crosslinked according to their crosslinking strategy. The obtained samples were stored in 6-well plates containing 4 mL of HUVEC medium (+1% penicillin/streptomycin) per well in an incubator at 37 °C and 5% CO_2_. A media change took place three times per week. A total of 7 samples of a batch were measured on days 1, 4, 7 and 14 with MicroTester LT in HBSS at 37 °C. Due to the different stiffnesses of the hydrogels, a microbeam with a diameter of 0.3048 mm was used for Alg/HA/Gel ink. For ADA-GEL and GelMA, a microbeam with a diameter of 0.4064 mm was used according to the instructions of the MicroTester LT. The samples were loaded through two pressure cycles. For the first cycle, a deflection of 2% was chosen to avoid changes in the structure of the sample when determining the L_0_ value. For the second cycle, a deflection of 10% was chosen to determine the Young’s modulus. From the calculated stress-strain curve, the slope was determined in the range of 2–5% to obtain the Young’s modulus of the hydrogels. The MicroTester LT experiments were performed with 7 samples from one batch per day.

### 2.11. Degradation Behavior

Samples for the degradation study were obtained from a silicone mold with cylindrical recesses with a diameter of 7 mm. An amount of 300 μL of each of the inks was added to the holes and then crosslinked. The samples were subsequently weighed to obtain the initial weight. The samples were stored in the incubator in 6-well plates in sterile Falcon 100 μm Cell Strainer (Corning, New York, NY, USA) and HUVEC medium (+1% penicillin/streptomycin). The weight of the samples was determined on days 1, 4, 7, and 14. The degradation study results were determined from two ink batches of 5 samples each. This resulted in at least 10 technical replicates on days 0, 1, 4, 7, and 14. The weight percentage of the samples was determined using Equation (1). W_0_ represents the starting weight of the sample, which was determined on day 0. W_t_ denotes the weight of the day of measurement, similar to other studies [31].

(1) $$W_{r}=\left(\frac{W_{t}}{W_{0}}\right)\cdot 100$$

### 2.12. Statistics

Statistical analysis was performed using GraphPad Prism 8.1.2 (GraphPad Software, La Jolla, CA, USA). Differences between groups were analyzed using the following tests: Shapiro-Wilk normality test, followed by ANOVA and Tukey multiple comparisons test. An ANOVA and Kruskal-Wallis multiple comparison test were performed to evaluate if differences between the Young’s moduli were statistically significant. The significant *p*-value was set to ≤0.05.

## 3. Results

### 3.1. Printability

**Figure 3:**
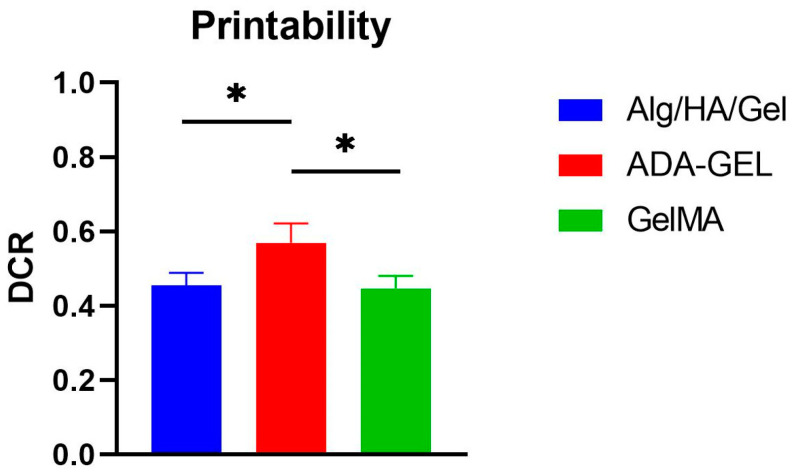
DCR of the three hydrogels Alg/HA/Gel, ADA-GEL, and GelMA directly after printing. Data represented as mean ± standard deviations. * *p* < 0.05.

As shown in Figure 3, good printability could be determined for all three hydrogels. For GelMA, the lowest diagonal cross ratio was found to be 0.44 (±0.04). A statistically non-significantly higher ratio was found for the Alg/HA/Gel ink mixture with 0.46 (±0.04). The best printability result was obtained for ADA-GEL, with a statistically significant higher DCR of 0.57 (±0.05) compared with the values of the other two inks.

### 3.2. Rheometer Analysis

**Figure 4:**
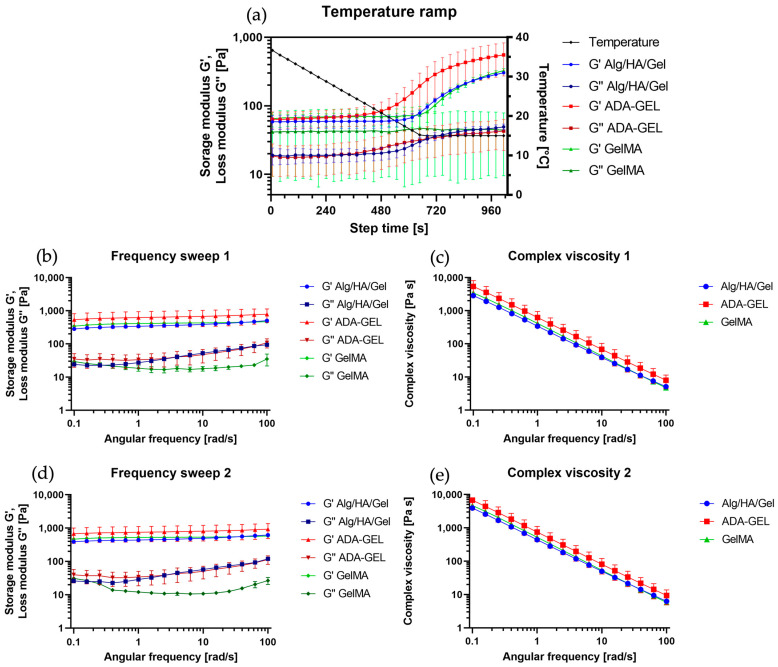
(**a**) Temperature ramps of the three measured hydrogels. From the initial temperature of 37 °C the inks were cooled to 15 °C, which was maintained for 6 min. Logarithmic representation of the left *y*-axis. (**b**) Frequency sweep 1 directly after the sample was cooled down to 15 °C. G′ represents storage modulus of the respective gel, and G″ the loss modulus. (**c**) Value of omplex viscosity 1, coinciding with frequency sweep 1. (**d**) Frequency sweep 2: 10 min after frequency sweep 1. G′ represents storage modulus of the respective gel, and G″ the loss modulus. (**e**) Value of complex viscosity 2, coinciding with frequency sweep 2. Double logarithmic plot of the axes at (**b**–**e**). Data represented as mean ± standard deviations.

The measured temperature ramp of the hydrogels is shown in Figure 4a. The storage modulus G′ was always higher than the loss modulus G″ for all inks. During the cooling process, the gelation of the inks was evident from the increase in storage modulus G′. For ADA-GEL and Alg/HA/Gel, the increase in storage modulus could be observed even before 15 °C was reached. For ADA-GEL, it was observed at approx. 19 °C and for Alg/HA/Gel at approx. 17 °C.

Figure 4b,d show the behavior of the gels at a constant deformation of 1% and increasing frequency in the range of 0.1–100 rad/s (0.016–16 Hz). G′ was always higher than G″, confirming the gel-like behavior of the inks. At 15 °C, the hydrogels behaved like viscoelastic solids. Between frequency sweep 1 and frequency sweep 2, which were measured at 10-min intervals, a small upward shift can be observed. This can be attributed to the fact that the hydrogels continued to gel during this period and had not yet reached their equilibrium state at frequency sweep 1, which is already evident from Figure 4a since no plateau was reached. The amplitude sweeps performed confirmed that the measurement of the frequency sweeps was carried out in the linear range.

Furthermore, the complex viscosities of the hydrogels are shown in Figure 4c,e. The shear-thinning behavior with increasing frequency was clearly evident. Comparing the complex viscosity 1 with the complex viscosity 2, which was recorded together with the frequency sweep 2 (10 min later), a small upward shift can be seen for each hydrogel in the entire frequency range.

### 3.3. MicroTester LT Measurements

**Figure 5:**
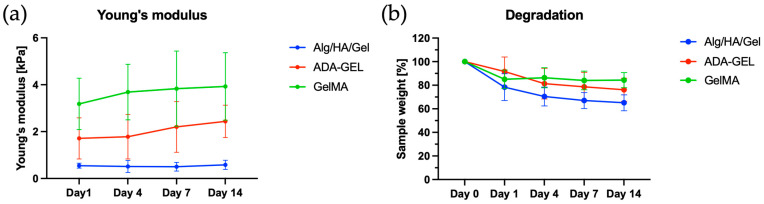
(**a**) Young’s moduli of the three hydrogels: Alg/HA/Gel, ADA-GEL, and GelMA. (**b**) Degradation behavior of the three hydrogels: Alg/HA/Gel, ADA-GEL, and GelMA. Data represented as mean ± standard deviations.

Comparing the samples prepared from Alg/HA/Gel with each other over the days, no significant differences in the Young’s moduli were observed (Table 1). Similarly, the measured Young’s moduli of the ADA-GEL samples over days 1 to 14 showed no statistically significant differences. The same could be detected for GelMA (Figure 5a). However, when comparing the inks with each other, statistically significant differences could be observed. For GelMA, a statistically significant higher Young’s modulus could be measured on day 1 than for the Alg/HA/Gel blend. Similarly, the Young’s modulus of Alg/HA/Gel was also statistically significantly lower than that of ADA-GEL. Moreover, on day 4, a statistically significant higher Young’s modulus could be measured for GelMA and ADA-GEL than for Alg/HA/Gel. On day 4, the Young’s modulus measured for GelMA was also statistically significantly higher than that for ADA-GEL. On day 7, the Young’s modulus of GelMA and ADA-GEL was statistically significantly higher than that of Alg/HA/Gel. On day 14, only a statistically significant higher value of GelMA could be measured compared with the Young’s modulus of Alg/HA/Gel. It appeared that GelMA was the stiffest gel, whereas Alg/HA/Gel was the softest. The Young’s modulus of ADA-GEL was between the values of GelMA and Alg/HA/Gel on all days considered.

### 3.4. Degradation Behavior

After 24 h, degradation was observed for all hydrogels (Table 2, Figure 5b). For the Alg/HA/Gel ink mixture, a statistically significant loss of mass from 100% to 78.39 ± 11.33% was observed on day 1. Further loss of mass was observed on day 7 for Alg/HA/Gel. At day 14, a statistically significant lower weight percentage was observed for Alg/HA/Gel compared with day 0. On day 1, a loss of mass from 100% to 91.61 ± 12.36% was observed for ADA-GEL. Furthermore, for ADA-GEL, a statistically significant weight loss was detected at day 4 compared with day 1. Further loss of mass was observed on day 7 for ADA-GEL. A statistically significant lower percentage was observed for ADA-GEL on day 14 compared with day 0. Significant degradation to 85.07 ± 7.826% was observed for the samples produced from GelMA ink on day 1 compared with day 0. GelMA showed a slight statistically insignificant increase in mass on day 4 compared with days 0 and 1. On day 7, GelMA lost only slightly more mass. The weight percentage obtained for GelMA at day 14 was statistically significantly lower than that obtained at day 0.

Compared with the Alg/HA/Gel ink, the value recorded for ADA-GEL was statistically significantly higher at day 1. The values of ADA-GEL and GelMA were statistically significantly higher than the weight percentage of Alg/HA/Gel samples on days 4, 7, and 14. For Alg/HA/Gel, a total mass loss of 34.87% was detected over 14 days. For ADA-GEL, a total mass loss of 23.82% was observed over 14 days. For GelMA, only a statistically significant mass loss of 14.93% from day 0 to day 1 could be detected. The total mass remained almost the same over the other days.

### 3.5. Single Cells

#### 3.5.1. Day 1 (Single Cells)

**Figure 6:**
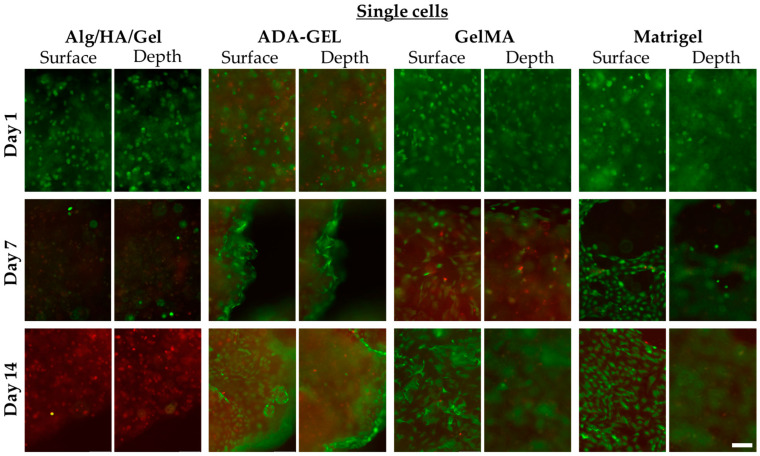
Single cells on days 1, 7, and 14 in Alg/HA/Gel, ADA-GEL, GelMA, and Matrigel on the surface of the scaffolds and at 100 μm depth. Green = live cells, red = dead cells; 10× magnification. Scale bar = 100 μm.

**Figure 7:**
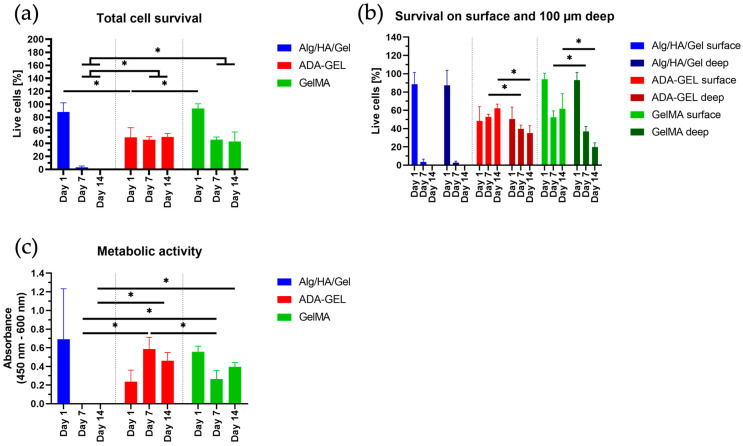
(**a**) Total cell survival in each bioink. (**b**) Proportion of live cells on the surface and live cells at 100 μm depth of the gels. (**c**) Metabolic activity of the single cells in the bioinks. Data represented as mean ± standard deviations. * *p* < 0.05.

As shown in Figure 6, a large number of viable cells could be identified in Alg/HA/Gel on day 1. For day 1, the survival rate was 88.27% in Alg/HA/Gel out of a total of 2442 cells counted (in 3 × 3 ROIs with 2 planes) (Figure 6). In ADA-GEL, compared with Alg/HA/Gel a considerable number of dead cells could already be detected on day 1. In contrast, the survival rate was 49.39% in ADA-GEL, with a total number of 1858 cells counted (in 3 × 3 ROIs with 2 planes). On the first day, the survival rate of 2007 counted cells in GelMA was 93.43%. After one day, the largest number of living cells could be detected in GelMA. The survival rate was statistically significantly higher in GelMA and Alg/HA/Gel than in ADA-GEL, as shown in Figure 7a. As can be seen in Figure 7b, none of the bioinks showed a difference in living cells between the surface and the 100 μm depth on day 1. Moreover, in Matrigel, a large number of living cells could be observed on day 1.

With respect to the metabolic activity of the single cells, no statistically significant difference between the gels could be detected on day 1 (Figure 7c).

#### 3.5.2. Day 7 (Single Cells)

After 7 days, most of the printed cells had died in Alg/HA/Gel, and hardly any living cells were identified, as shown in Figure 6. The survival rate was 3.07% out of a total of 2823 cells counted. In ADA-GEL, the live/dead ratio remained approximately the same as on day 1. The survival rate in ADA-GEL out of a total of 2391 cells counted was statistically significantly higher at 45.38% than in the Alg/HA/Gel bioink. A similar survival rate as in ADA-GEL was observed in GelMA for day 7. The survival rate in GelMA was 45.57% out of a total of 1785 cells counted. While no difference between the surface and the depth could be detected in the Alg/HA/Gel bioink, a considerable difference could be observed in the other two hydrogels. Statistically, significantly more live cells were observed on the surface of the bioinks. Cell-cell contacts have also been observed in individual cases. Almost the same behavior as in ADA-GEL and GelMA was observed in Matrigel.

The metabolic activity of single cells was statistically significantly higher in ADA-GEL than in the other two gels. No metabolic activity could be detected in Alg/HA/Gel, which is consistent with the observation of the survival rate assay. It was still statistically significantly higher in GelMA than in Alg/HA/Gel.

#### 3.5.3. Day 14 (Single Cells)

**Figure 8:**
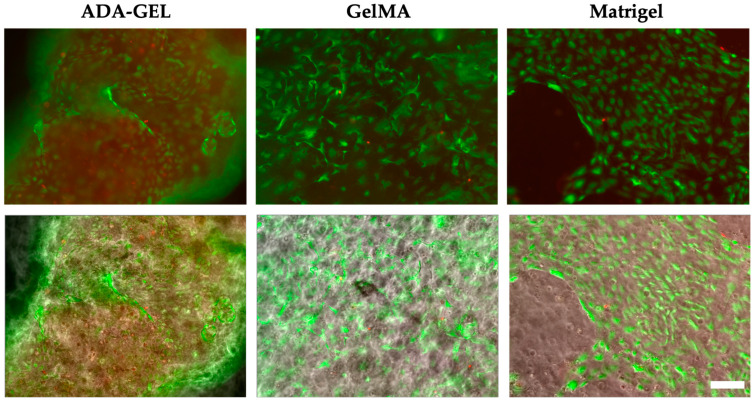
Example images of single cells at day 14, showing cell-cell contacts at the surface of the scaffolds in ADA-GEL, GelMA, and Matrigel. Upper image: live (green) and dead (red), lower image merges with phase contrast. Scale bar = 100 μm.

On day 14 in Alg/HA/Gel, no more living cells could be detected out of a total of 3862 cells counted. In contrast, a slight statistically insignificant increase in the live cell ratio was also observed in ADA-GEL out of a total of 2411 cells counted on day 14. In GelMA, the total cell survival remained the same out of a total of 1839 cells counted, and no major difference could be detected compared with day 7 (Figure 7a). With respect to the surface of GelMA and ADA-GEL, the number of living cells was statistically significantly higher there than inside the gels (Figure 7b). Both scaffolds were almost completely covered by the cells, and increased cell-cell contacts could be detected (Figure 8). Moreover, in Matrigel, a higher number of cell-cell contacts could be detected on the surface of the gel on day 14 than on day 7. Alignment of the cells could be detected for the gels ADA-GEL, GelMA, and Matrigel.

Metabolic activity could be detected for ADA-GEL and GelMA. Since there were no living cells left in Alg/HA/Gel, no metabolic activity was measured. Therefore, metabolic activity was also statistically significantly higher in ADA-GEL and GelMA bioinks than in Alg/HA/Gel at day 14 (Figure 7c). On day 14, a trend for higher metabolic activity was detected in GelMA compared with day 7, due to the fact that the cells proliferated at the surface and an increased number of cell-cell contacts was observed.

### 3.6. Spheroids

#### 3.6.1. Day 0 (Spheroids)

**Figure 9:**
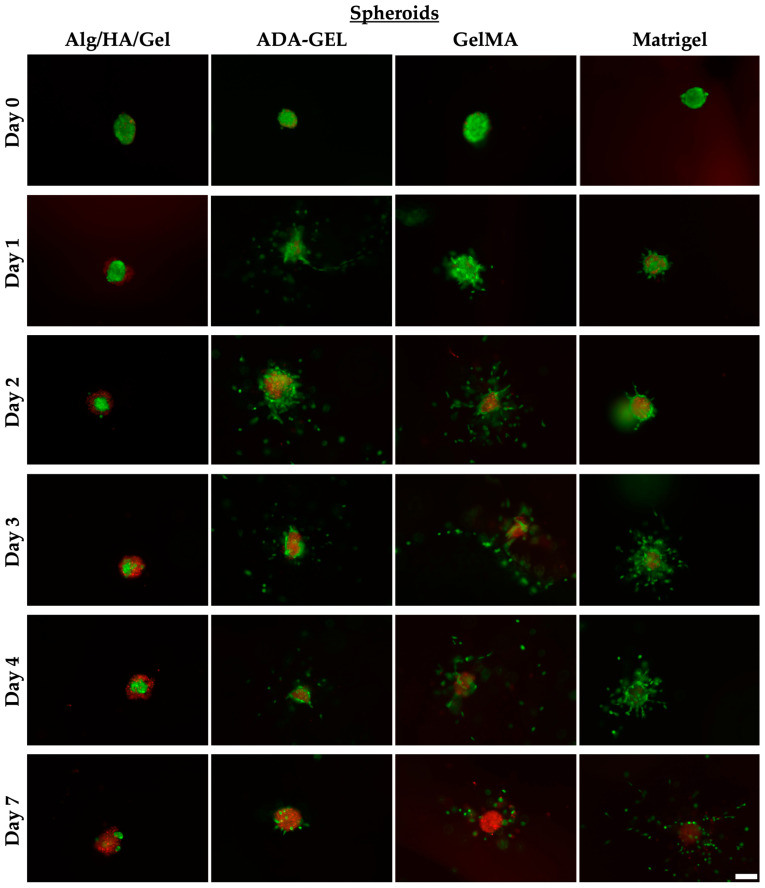
Spheroids on days 0, 1, 2, 3, 4, and 7 embedded in Alg/HA/Gel, ADA-GEL, GelMA, and Matrigel. Green = live cells, red = dead cells; 10× magnification. Scale bar = 100 µm.

On day 0, all spheroids had an almost identical appearance (Figure 9). The outer shell was green, which characterized the living cells, and some dead, red-stained cell nuclei appeared slightly through the green shell in the hydrogels Alg/HA/Gel, ADA-GEL, and GelMA. Only in the case of spheroids printed in Matrigel could no red (dead) component be identified.

#### 3.6.2. Day 1 (Spheroids)

**Figure 10:**
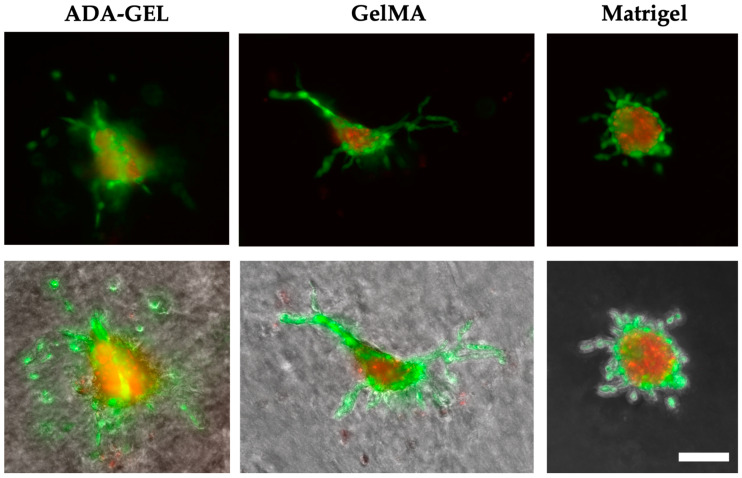
Example images for visible sprouting in ADA-GEL, GelMA, and Matrigel. Upper image: live (green) and dead (red), lower image merges with phase contrast. Scale bar = 100 μm.

**Figure 11:**
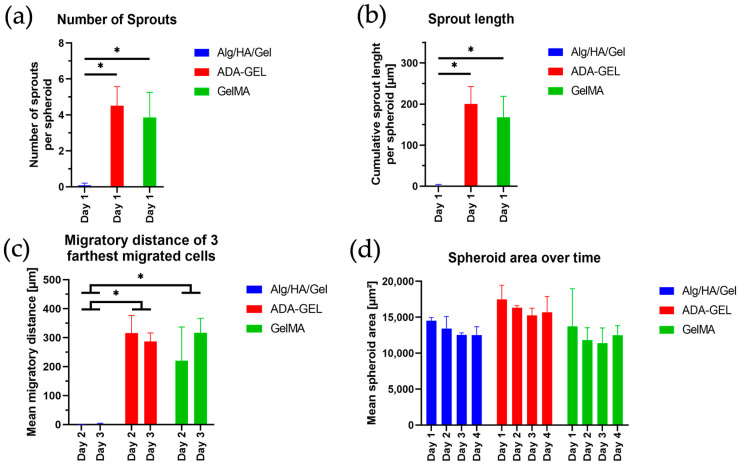
(**a**) Number of sprouts formed in the hydrogels on day 1, (**b**) Length of sprouts formed in the gels, (**c**) Migration distance of the three farthest migrated cells on days 2 and 3, (**d**) Spheroid area over days 1, 2, 3, and 4. Data represented as mean ± standard deviations. * *p* < 0.05.

The spheroids printed in Alg/HA/Gel showed, from day 1, as seen in Figure 9, a live green core in the center surrounded by dead cells. Almost no sprouting or migration of the spheroids could be observed compared with the other bioinks, despite the addition of PMA to stimulate sprouting and migration. In the spheroids printed in ADA-GEL sprouts, this could already be observed on the first day (Figure 10). Sporadically migrated cells were also observed as early as day 1. The sprouts formed in ADA-GEL tended to be the longest compared with the other inks (Figure 11a,b). The spheroids in GelMA showed similar behavior to the colonies printed in ADA-GEL on the first day. Sprout formation was clearly visible (Figure 10). As with the ADA-GEL, sporadic migration of cells could also be detected on the first day. The number of sprouts and their length were not significantly lower in GelMA than in ADA-GEL but were, in contrast, statistically significantly higher than in Alg/HA/Gel. On the first day, sprouting of the spheroids could be detected in Matrigel as well. However, migrated cells could not yet be observed on day 1, as in the ADA-GEL and GelMA hydrogels.

#### 3.6.3. Day 2 (Spheroids)

On day 2, virtually no more migrating cells were observed, and the green living core in the center of the spheroid became progressively smaller compared with day 1 (Figure 9) in Alg/HA/Gel. As shown in Figure 11c, on day two, a wide cell migration distance could already be observed in ADA-GEL as well as in GelMA. The formation of sprouts could hardly be detected on day 2 in ADA-GEL and GelMA; rather, the cells migrated away from the sprouts formed on day 1. The distance of the three farthest migrated cells was statistically significantly higher in ADA-GEL and GelMA than in Alg/HA/Gel, in which no cells migrated. However, sprouts were still detected in Matrigel on day 2. However, the migration of cells could not yet be observed in Matrigel after 2 days.

#### 3.6.4. Day 3 (Spheroids)

On day 3, the total area of the spheroid trended to decrease only slightly over time in Alg/HA/Gel (Figure 11d). The area changed little over time; rather, the cells in the colony died, with the red portion of the area increasing compared with the initial green core (Figure 9). On day 3, migration of further cells could be seen in ADA-GEL. The same behavior was observed in GelMA (Figure 9). A trending increase in cell migration distance from day 2 to day 3 was detected in GelMA. The migration distance of the three farthest cells was statistically significantly higher in ADA-GEL and GelMA than in Alg/HA/Gel, since no migration was detected there on day 3 (Figure 11c). The spheroids embedded in Matrigel showed a high number of migrated cells around the core on day 3. Only isolated sprouts were still visible on the spheroid core. The spheroidal area of ADA-GEL and GelMA decreased over the days, as did that of Alg/HA/Gel. However, no statistically significant change could be observed here either (Figure 11d).

#### 3.6.5. Day 4 (Spheroids)

On day 4, no major change in the spheroids could be observed in Alg/HA/Gel compared with day 3 (Figure 9). The migration of single cells continued to increase over time in ADA-GEL, GelMA, and Matrigel, and fewer migrated cells were observed around the initial core area. In terms of spheroid area, no statistically significant difference was found in any of the bioinks (Figure 11d).

#### 3.6.6. Day 7 (Spheroids)

On day 7, similar morphology was detected in all spheroids. After 7 days, the dead core of the spheroid remained (Figure 9). While in the Alg/HA/Gel bioink, the initially living cells died slowly in the core, in the other hydrogels, most of the living cells had migrated away. In Matrigel, the largest number of migrated cells could be detected around the spheroid core. In ADA-GEL and GelMA, in which the migration of the cells started a day earlier, the cells had already migrated further away from the spheroid core.

## 4. Discussion

In the present study, endothelial cells embedded in the three different hydrogels Alg/HA/Gel, ADA-GEL, and GelMA were investigated in detail with respect to cell survival, sprouting, migration, and proliferation. For this purpose, cells were printed as single cells as well as encapsulated in the form of a spheroid in the inks to shed light on the suitability of the hydrogels for angiogenesis. The behavior of the cells in the inks was additionally compared with that of the standard material, Matrigel. Matrigel is a basement membrane matrix derived from mouse sarcoma and is considered a standard material for many cell culture applications. However, the application of Matrigel is limited due to its complex, poorly defined, and highly variable composition and insufficient shape fidelity for bioprinting [7,32]. While the behavior of endothelial cells in GelMA has been described many times in the literature, their behavior in the other two hydrogels in the context of bioprinting has not been studied in such detail in previous research [33].

Sufficiently good printing precision and shape fidelity were determined for the inks Alg/HA/Gel, ADA-GEL, and GelMA. For ADA-GEL, the highest DCR was observed with 0.57 (± 0.05). In a study by Bednarzig et al. (2022) [30], a high DCR of about 0.75 could be determined for an alginate hydrogel with bioactive glass as filler. Whereas in another study by Heid et al. (2022) [34], a DCR of 0.5 was obtained for an ADA-GEL in combination with bioactive organic fillers. The good printability of ADA-GEL can mainly be attributed to the pre-crosslinking, which increases the shape fidelity significantly [24]. Although the DCRs determined for the inks are good, they can be further improved in the future by adding, for example, organic fillers, leading to a more precise result. The oscillatory measurements demonstrated the shear-thinning behavior of the inks. Shear-thinning behavior reduces the viscosity of the inks under load and returns to a more viscous state when unloaded, which can support shape fidelity [35,36]. Therefore, shear-thinning behavior is particularly important for extrusion-based printing, as the higher shear rates in the printing needle during extrusion facilitate better deposition of the hydrogel [35]. Furthermore, a disadvantage of extrusion-based bioprinting is that cell survival decreases when higher pressures or needles with smaller diameters are used, and there is no shear-thinning behavior of the material [36,37,38].

Based on the frequency sweeps performed, the gel-like material behavior of the hydrogels at 15 °C could also be demonstrated, as G′ was larger than G″ at all frequencies measured. Since the printing took place directly after the cooling process, the determined DCR values refer to the first frequency sweep. The small upward shift that occurred from viscosity 1 to viscosity 2, which was observed for all hydrogels, can be attributed to the progressive gelation of the hydrogels. In ADA-GEL, due to the higher polymer content in the ink compared with the others, the viscosity was the highest. For all the inks, G′ was much higher than G″ at the printing temperature, indicating good shape fidelity temporarily after deposition. Based on these results, it can be assumed that all inks are optimally suited for bioprinting.

When evaluating the MicroTester LT measurements, the lowest elastic modulus was observed for Alg/HA/Gel. This can be attributed to the fact that the alginate concentration of the Alg/HA/Gel ink, which is responsible for the crosslink density, was relatively low. Increasing the alginate content also increased the stiffness greatly in the study by Schmid et al. (2021) [7]. This allows the ink to be customized.

The stiffness observed for ADA-GEL was 1.72 ± 0.88 kPa on day 1 and 2.44 ± 0.69 kPa on day 14. Crosslinking of gelatin with mTG leads to a significantly higher elastic modulus of ADA-GEL compared with Alg/HA/Gel, as this strengthens the hydrogel matrix [26]. Moreover, the alginate concentration in ADA-GEL is significantly higher than in the Alg/HA/Gel ink. By using mTG in different concentrations for crosslinking, the stiffness can be adjusted [26]. The increase in stiffness observed in ADA-GEL over days could be due to the release of non-crosslinked gelatin [26]. Due to the temperature increase during incubation from room temperature (22 °C) to 37 °C, further crosslinking of the gelatin with the remaining mTG could occur. This would lead to an increase in stiffness. It was also reported in a past study that an increase in pH causes crosslinking of mTG. The constructs formed were much more stable at a pH of 6 than at a more acidic pH [39]. Therefore, it can be assumed that the transfer of the constructs into the cell culture medium at a pH of about 7 resulted in further crosslinking of the gelatin with the residual amounts of mTG contained in ADA-GEL over time. This could explain an increase in stiffness in ADA-GEL.

For GelMA, the highest Young’s modulus was measured within the study. Similar values for elasticity as measured in this study have also been measured in the past for hydrogels made from 5% GelMA. Furthermore, it was shown in the study by Wu et al. (2019) [40] that increasing the GelMA concentration to 10% also resulted in 10-fold higher elastic moduli.

For soft tissue, stiffness values of 1 kPa (liver) [41,42] to 8 kPa (myocardium, kidney) can be found in the literature. For the upper skin layer, a Young’s modulus of 0.11 kPa was measured. In contrast, the reticular dermis showed a much higher Young’s modulus of 160 kPa [43].

Stiffness has been shown in the past to be a regulator of cell behavior and adhesion. Compliant substrates have been shown to promote network formation and tubulogenesis in endothelial cells [44]. It was observed by Saunders et al. (2010) [45] that HUVECs formed 2D networks on inert polyacrylamide gels with different Young’s moduli and on soft gels with Young’s moduli of 0.14 kPa and 0.675 kPa. In contrast, cell spreading rather than stable network formation was observed at higher Young’s moduli in the range of 1.05 to 2.5 kPa. This was attributed to the fact that cells on softer substrates formed fewer cell-substrate adhesions, and therefore cell-cell adhesion predominated [45]. Thus, the directed migration of HUVECs into the tissue is supported by a sufficient stiffness gradient. In contrast, too much stiffness (above 4 kPa) acts as a mechanical barrier, which negatively affects migration and may even completely prevent network formation. Similarly, tube formation can be prevented by having a stiffness that is too low. Therefore, stiffnesses in the range of 0.5 to 2.5 kPa lead to increased migration and also to an increase in sprout formation [46]. Hence, the stiffnesses measured in this study are all in the above range (0.5–2.5 kPa and below 4 kPa), which has been identified in previous studies as the stiffness range that reconciles migration and adhesion [46].

In terms of degradation behavior, a slow mass loss over 14 days was observed for all gels. This demonstrated the successful crosslinking of the hydrogels. Suitable crosslinking strategies are of utmost relevance, especially for hydrogels, as their rapid degradation is an obstacle for many applications.

For alginate-based hydrogels, calcium is usually used for crosslinking. However, crosslinking with barium has also historically resulted in a structure with high strength and a slower release of gelatin. Cell-toxic effects of barium were not observed [47]. Furthermore, it was reported that the degradation rate of ADA-GEL can be adjusted by the addition of mTG at different concentrations [26]. The additional crosslinking of Alg/HA/Gel with mTG could lead to slower degradation of Alg/HA/Gel in addition to the increase in stiffness. This would allow adjustment of the degradation behavior of the Alg/HA/Gel blend. Additionally, crosslinking with mTG could lead to an increase in stiffness.

For the degradation behavior of GelMA, a similar mass loss of 18.70% was observed after 14 days in a study by Heltmann-Meyer et al. (2021) [11]. It can be concluded that UV crosslinking of GelMA results in stable constructs that degrade slowly [48]. Due to its slow degradation, GelMA can be used as an ink for long-term applications such as drug-derived tissue containers or bone tissue engineering, in addition to vascular tissue engineering applications [11,49,50]. Long-term applications may require biomaterials that degrade slowly (over months to years) while promoting angiogenesis [11]. For cartilage regeneration, a period of several weeks is sufficient for cartilage cells to establish their own ECM. The degradation behavior of the inks Alg/HA/Gel, ADA-GEL, and GelMA investigated here is therefore well controllable and can be tailored to the corresponding applications.

Comparing the three printed inks, cells migrate and survive well in the ADA-GEL and GelMA hydrogels, whereas none of the above behaviors could be detected in the Alg/HA/Gel. This suggests that the ink originally developed for tumor cells is not a suitable environment for the network formation of endothelial cells. Although high biocompatibility and good cell survival were demonstrated by Schmid et al. (2021) [7] also for immortalized adipose-derived mesenchymal stem cells (ADSCs) printed in the ink, this could not be confirmed for the HUVEC cell line in the Alg/HA/Gel ink. Survival of tumor cells could also be demonstrated in the Alg/HA/Gel ink by Schmid et al. (2021) [7]. However, unlike tumor cells, HUVECs cannot proliferate from single cells. Furthermore, HUVEC cells cannot form multicellular structures to orient themselves due to their polarity. Additionally, the cells cannot migrate through the ink. The death of HUVEC cells in the ink can also be attributed to the amount of alginate, which is characterized by insufficient cell-material interaction and inefficient cell attachment.

Looking at the single cells from day 7 on, a large number of dead cells were observed in the Alg/HA/Gel, which increased over time. The number of dead cells at the outer edge of the spheroid from day 1 indicates that the ink does not provide a suitable environment for the survival of the cells within the hydrogel. In ADA-GEL, the alginate is present as alginate dialdehyde, which has been covalently crosslinked. In previous studies, it was shown that pre-crosslinked alginate has favorable viscoelastic properties that could facilitate cell migration [51]. Therefore, the disadvantages of alginate can be overcome by partial oxidation and pre-crosslinking, so that ADA-GEL provides a better environment for HUVECs than normal alginate, ADA-GEL, or Alg/HA/Gel [9]. Hence, the pre-crosslinked ADA-GEL used in this study supposedly offers significant advances over previously used ADA-GEL formulations.

Comparing the development of cells embedded in ADA-GEL and GelMA with that of HUVECs embedded in Matrigel, similar behavior was demonstrated. Cell survival and proliferation of the single cells were demonstrated for 14 days in ADA-GEL, GelMA, and Matrigel. The large number of dead cells that, in contrast to GelMA, were observed in ADA-GEL one day after printing could be due to the fact that the cartridge was filled with cells and ADA-GEL was additionally centrifuged at 872× *g* for 7 min before printing. This step was necessary when using the ADA-GEL to remove the bubbles from the gel for printing. It can be assumed that the centrifugal forces acting on the cells exert a negative influence on their survivability. It is unlikely that the mTG used to crosslink ADA-GEL resulted in increased cell death. Several studies have shown that the use of mTG even in higher concentrations does not lead to reduced cell viability [39,52,53,54]. However, in both ADA-GEL and GelMA, it was found that the endothelial cells grew preferably on the gel surface after 7 days, and cell-cell contacts could be observed on the surface. The high biocompatibility of the ADA-GEL and GelMA hydrogels was also demonstrated by the metabolic activity assay of cells printed on scaffolds over 14 days.

The migration of endothelial cells into the surrounding hydrogel, which is an important step for the formation of new blood vessels during angiogenesis, was further confirmed by the observation of spheroids over 7 days for ADA-GEL and GelMA [46]. The formation of spheroids allows cells to be packed tightly into a small volume, enhancing cell-cell interaction and thereby mimicking tissue architecture [55]. The hanging drop method was chosen for the preparation of spheroids because it can be used to produce a small number of spheroids with easily controllable sizes [56,57]. However, for the spheroids printed in the hydrogels, only a strong migration of cells and not yet the formation of networks could be detected.

On day 0, the spheroids in Matrigel are still particularly round and alive. No dead part is observed compared with the other hydrogels. This is likely because the lowest pressure was applied for bioprinting Matrigel. For the other three hydrogels, a much higher pressure was used. The higher pressure likely causes slight damage to the outer membrane due to deformation and shear forces. Thus, the inner, dead-red center of the spheroids can be seen to some extent. The round cells surrounding the spheroids observed from day 1 in ADA-GEL and GelMA suggest that tip cell migration occurred over time but was not followed by stalk cell migration. Filopodia-covered tip cells are important for sprout formation as they provide the direction for subsequent stalk cells. The stalk cells, which have a long and elongated morphology, are prevented from transforming themselves into tip cells by appropriate signaling cells [58,59]. The formation of stalk cells could no longer be detected in ADA-GEL and GelMA from day 2. Only in Matrigel was the formation of stalk cells observed until day 3. Migration of tip cells continued to increase over time in the three hydrogels, so that by day 7, cells dead in the original spheroid core remained. Those cells that were not already dead at the beginning migrated through the gel but did not yet align or form networks. This could underlie the time period, as previous research also observed better network formation of spheroids after 14 days than after 7 days [55]. In addition, this effect could be because significantly fewer spheroids were printed on the scaffolds, whereas single cells were present in large quantities throughout the scaffolds, presumably facilitating better cell-cell interaction between spheroids. Better cell-cell interaction could positively affect cell survival and network formation. In future research, the addition of higher numbers of spheroids to the hydrogels could increase cell-cell contacts and network formation.

The death of the endothelial cells inside the spheroid cannot be traced back to the printing process but occurs in spheroid cultures if they are not saved by survival factors such as VEGF and FGF-2. HUVEC spheroids form a two-compartment system. This consists of a surface monolayer with differentiated cells and a center with unorganized cells. The unorganized cells in the center die by apoptosis unless they are saved by survival factors. The cells inside are made susceptible to survival factors by cell-cell contacts, which enable spheroidal aggregation [60,61,62]. Although VEGF was contained in the medium, its concentration was not further investigated or altered in this study to ensure the survival of the unorganized cells in the spheroid center. Furthermore, the added VEGF concentration was also not known, as it was not disclosed by the manufacturer. Since the formation of tubes after 7 days was also not detected in spheroids printed in Matrigel, it cannot be assumed that the ADA-GEL and GelMA hydrogels lack, to some extent, the ability to promote angiogenesis. It is more likely that the amounts of additional important factors such as VEGF or bFGF that promoted angiogenesis were too low [33,61,63,64]. Migration may also be affected by low adhesion, resulting in membrane blebbing during migration [65]. Cell-matrix adhesion is also influenced by endothelial integrin receptors, which communicate with ECM proteins and mediate adhesion, regulating the proliferation and migration of stalk cells and tip cells.

Kretschmer et al. (2021) [46] reported, for example, that the presence of laminin, an ECM protein, is necessary for tube formation. Previous research has also shown that the addition of skin fibroblasts to HUVECs embedded in fibrin gel resulted in the formation of vascular networks with lumina, whereas in the absence of skin fibroblasts, endothelial cells migrated away and vessel formation was limited [66]. Therefore, the addition of skin fibroblasts could be interesting for future research. In a study by Bray et al. (2015) [67], it was also shown that cultivation of HUVECs in matrix metalloproteinase (MMP)-sensitive four-arm star-shaped poly(ethylene glycol) (starPEG)-heparin hydrogels resulted in tube formation only by the addition of arginylglycylaspartic acid and pro-angiogenic cytokines, supporting the hypothesis that vessel formation could occur in the ADA-GEL and GelMA hydrogels described here by the addition of angiogenic signals [67]. Similarly, another study by Ermis (2021), which cultured stem cells and HUVECs together in spheroids and embedded them in GelMA, demonstrated more pronounced network formation [55]. In a study by Rana D et al. (2022) [68], it was also shown that conjugation of VEGF to a 50-acrylate-modified aptamer encapsulated in GelMA together with HUVECs and human mesenchymal stromal cells (hMSCs) resulted in the controlled release of VEGF. Due to the controlled release, not only lumen-like microvascular networks but also a temporally controlled network organization could be detected. This study therefore also reconfirms the relevance of the addition of VEGF and its control for controlled network formation [22,68]. In a further study by Ruther et al. (2019) [20], a handmade double-needle extrusion system was used to produce a vascular construct that consisted of ADA-GEL in the outer layer and sacrificial gelatin in the inner layer. Cultivation with fibroblasts and endothelial cells confirmed migration over the entire period, but no capillary network could yet be confirmed [20]. Therefore, for the formation of larger vessels, the use of sacrificial inks could be useful in the future for the creation of cavities in the GelMA and ADA-GEL inks studied here [1].

The two promising pre-crosslinked inks, ADA-GEL and GelMA, should be further investigated for their angiogenic potential in the future due to their optimal mechanical properties and excellent biocompatibility. To achieve network formation, angiogenic factors should be added in sufficient concentration.

## 5. Conclusions

In this study, the three hydrogels Alg/HA/Gel, ADA-GEL, and GelMA were investigated for various aspects, and the survivability of HUVECs was compared with the standard material Matrigel. In terms of mechanical properties, good printability was recognized for all inks. The shear-thinning behavior of the inks was demonstrated. A suitable modulus of elasticity as well as tailorable degradation behavior were further observed. Therefore, it can be stated for all three investigated hydrogels that they are optimally suited for extrusion-based bioprinting due to their mechanical properties. The survival of HUVEC single cells up to 14 days and of spheroids up to 7 days in the GelMA and ADA-GEL bioinks was demonstrated. The proliferation and migration of HUVECs could be observed in the hydrogels as well. The migration and growth of the cells occurred preferentially on the scaffold surface. Similar behavior of HUVECs could be detected in the Matrigel. On day 7, cell-cell contacts and alignments of single cells were observed on the surfaces of GelMA, ADA-GEL, and Matrigel. In the case of the spheroids, a strong cell migration through the hydrogels towards the surface could be observed over 7 days. In the Alg/HA/Gel bioink, the endothelial cells survived neither as single cells nor as spheroids. Even though good mechanical properties could be determined for all inks, the Alg/HA/Gel bioink proved to be unsuitable for vascular tissue engineering in the cell study. It can be concluded that the ADA-GEL and GelMA bioinks offer promising matrices for vascular tissue engineering.

## Figures and Tables

**Table 1:** Young’s modulus of the three hydrogels Alg/HA/Gel, ADA-GEL, and GelMA on days 1, 4, 7, and 14.

Day	Young’s Modulus [kPa]		
	Alg/HA/Gel	ADA-GEL	GelMA
1	0.55 ± 0.11	1.71 ± 0.89	3.18 ± 1.1
4	0.51 ± 0.26	1.78 ± 0.95	3.69 ± 1.19
7	0.50 ± 0.183	2.20 ± 1.08	3.83 ± 1.61
14	0.58 ± 0.19	2.44 ± 0.69	3.93 ± 1.44

**Table 2:** Degradation behavior of the three hydrogels Alg/HA/Gel, ADA-GEL, and GelMA on days 0, 1, 4, 7, and 14.

Day	Sample Weight [%]		
	Alg/HA/Gel	ADA-GEL	GelMA
0	100	100	100
1	78.39 ± 11.33	91.61 ± 12.36	85.07 ± 7.826
4	70.48 ± 8.02	81.32 ± 13.02	86.35 ± 8.46
7	67.00 ± 6.76	78.68 ± 13.31	84.07 ± 7.87
14	65.13 ± 6.75	76.18 ± 9.80	84.33 ± 6.44

## Data Availability

Data available on request.
